# Data for the effects of ER and Golgi stresses on the ER–Golgi SNARE Syntaxin5 expression and on the βAPP processing in cultured hippocampal neurons

**DOI:** 10.1016/j.dib.2015.08.023

**Published:** 2015-09-04

**Authors:** Kei Suga, Ayako Saito, Tatsuya Mishima, Kimio Akagawa

**Affiliations:** Department of Cell Physiology, Kyorin University School of Medicine, Mitaka, Tokyo 181-8611, Japan

## Abstract

This paper reports the data for the effects of organelle stresses on the ER–Golgi-soluble *N*-ethylmaleimide-sensitive factor-attachment protein receptors (ER–Golgi SNAREs) syntaxin 5 (Syx5) in neuronal cells. Quantitative as well as qualitative data are presented here to verify the upregulation of Syntaxin 5 (Syx5) under ER and Golgi stresses in hippocampal neurons. Changes in the processing of β-amyloid precursor protein (βAPP) under ER stress were analyzed by immunological assays. In addition, our data shows the specific increase of Syx5 expression under ER and Golgi stresses. Interpretation of our data and further extensive insights into the role of Syx5 in βAPP processing under organelle stress can be found in "ER and Golgi stresses increase ER-Golgi SNARE Syntaxin5: Implications for organelle stress and βAPP processing" [1].

**Specifications table**

**Table:** 

Subject area	*Biology*

More specific subject area	*Stress response, neuronal survival, ER–Golgi SNAREs, syntaxin*
Type of data	*Text file, graph, figure*
How data was acquired	*Western blotting, immunofluorescence, microscopy, luminometer, ELISA*
Data format	*Analyzed*
Experimental factors	*Rat hippocampal culture neurons were treated with ER or Golgi stress inducers*
Experimental features	*ER and Golgi stress treated cells were processed for western blotting, immunocytochemical, calcium imaging, and viability assays.*
Data source location	*Kyorin University School of Medicine, Tokyo, Japan*
Data accessibility	*All data are provided in this article*

**Value of the data**

• Data show the effects various stress inducers on the expression and intracellular localization of ER–Golgi SNARE Syx5 in hippocampal neurons.
• Data obtained by immunological assays show the effects of ER stress inducers on the βAPP processing in primary culture neurons.
• The data provided here may inform many researchers that are investigating neurodegenerative diseases of the effect of organelle stress in mammalian cells.

## 1 Data, experimental design, materials and methods

**Fig. 1:**
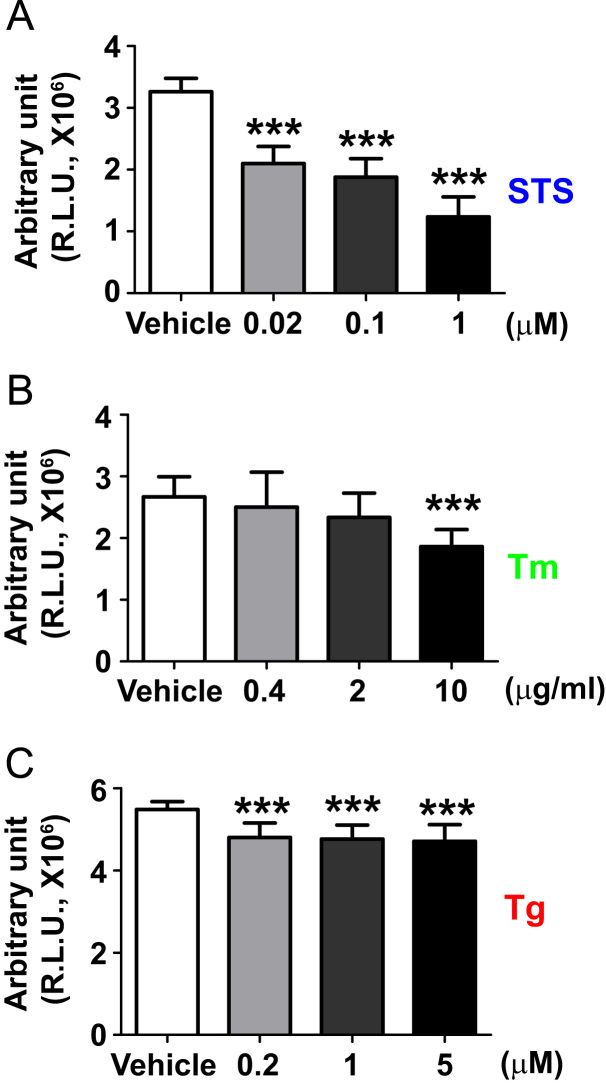
Cell viability of 14 DIV hippocampal culture neurons.

**Fig. 2:**
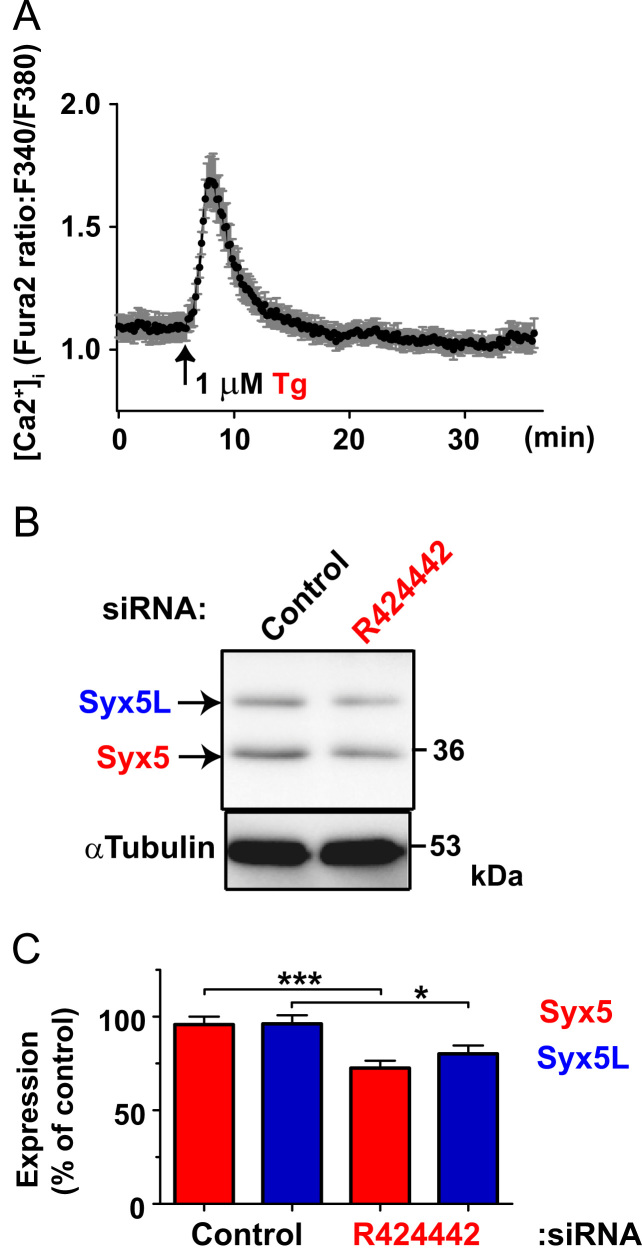
Effects of Tg and siRNA for Syx5 on hippocampal neurons.

**Fig. 3:**
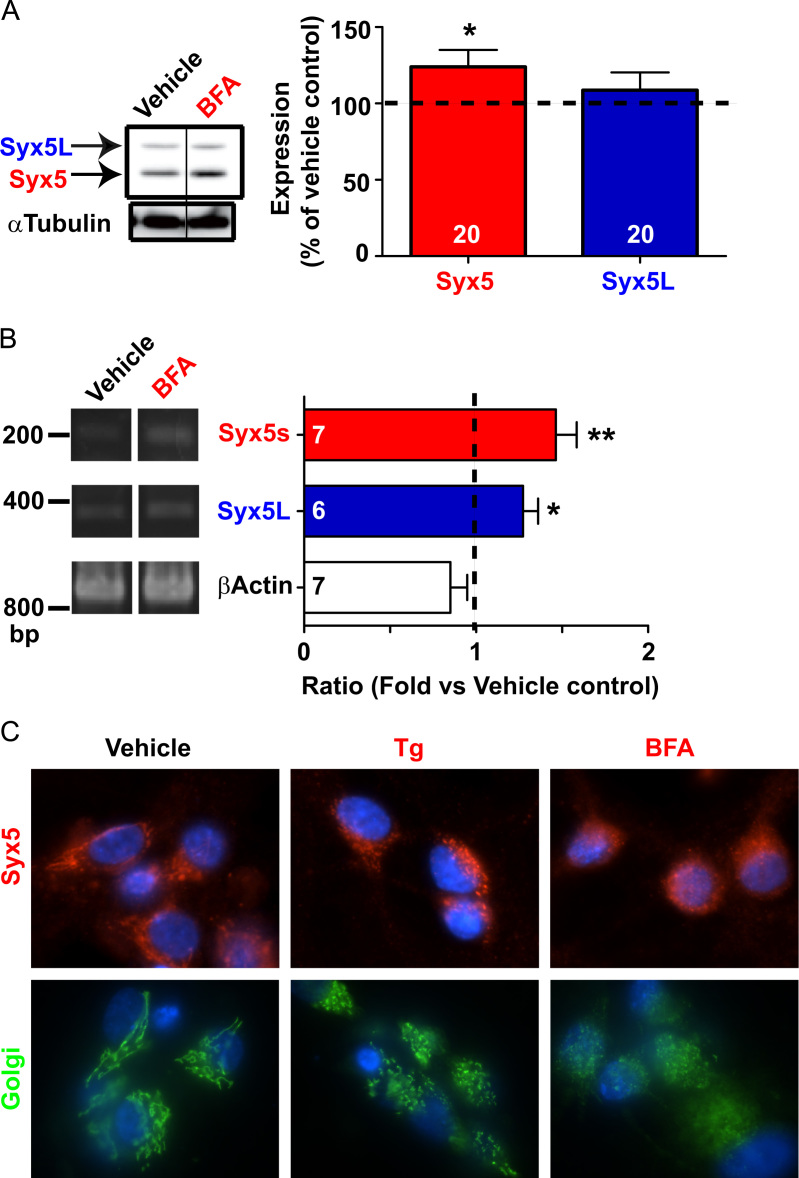
BFA increases Syx5 expression and induces fragmentation of the Golgi.

**Fig. 4:**
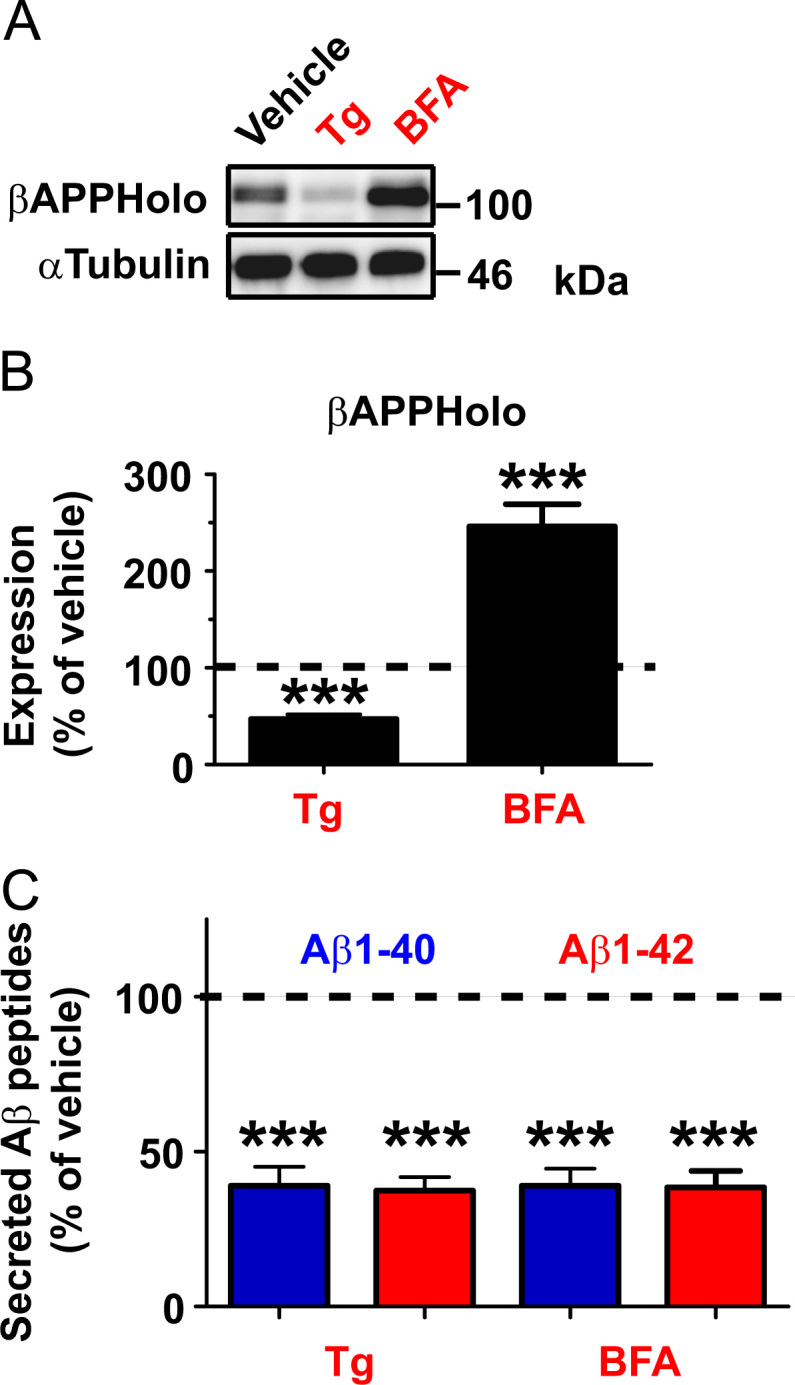
ER stress reduces Aβ peptide secretion in hippocampal neuron.

**Fig. 5:**
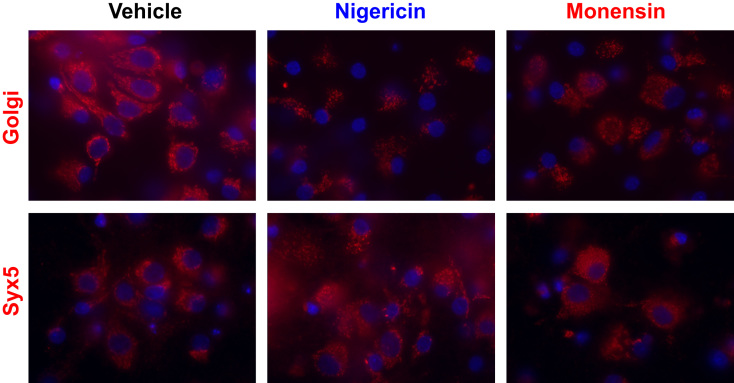
Effects of nigericin and monensin on the morphology of Golgi apparatus and the distribution of Syx5 in hippocampal culture neurons.

**Fig. 6:**
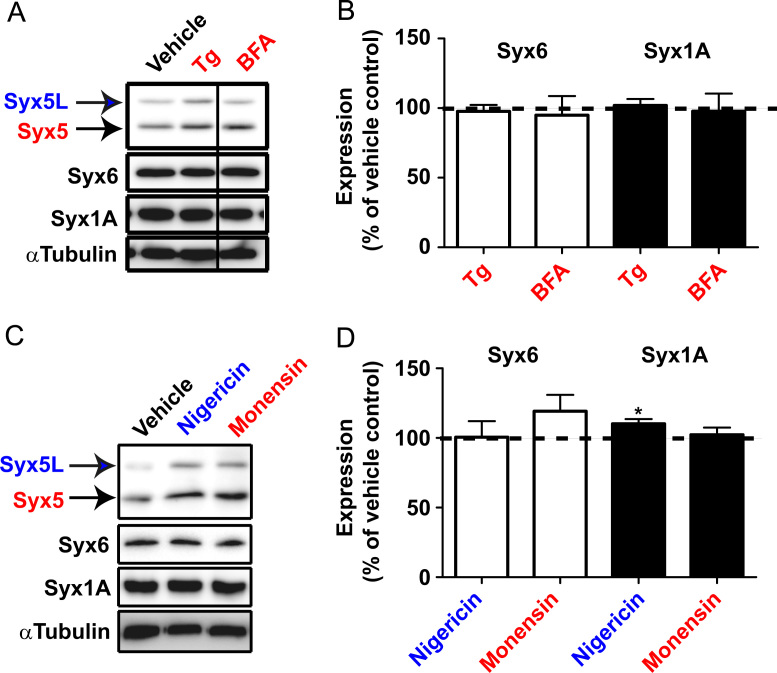
Specificities of Syxs in response to ER and Golgi stresses.

Here we showed quantitative as well as qualitative data concerning the effects of various ER and Golgi stress inducers on the expression and the localization of ER–Golgi SNARE proteins in hippocampal neurons. The data presented here provides important information on the use of various toxins that induce organelle stress in primary culture neurons. Syx5 is a member of ER–Golgi SNAREs and is a major player in the transport processes in the early secretory compartment [2]. Moreover, we have previously shown that Syx5 may have a role in modulating βAPP processing under ER stress [3]. However, the roles of Syx5 under Golgi stress in neurons are poorly understood. Here, we used rat hippocampal neurons to elucidate the mechanism of Syx5 upregulation under organelle stress by biochemical, immunocytochemical, and imaging analyses. We confirmed the effect of stress-inducing reagents on cell viability, and the effect of siRNA on the expression of Syx5 proteins in culture neurons (Figs. 1 and 2) using western blotting and luminescent assays. By RT-PCR and western blotting analyses, we also demonstrated the effect of other ER stress inducer brefeldin A (BFA) on the amount of Syx5 expression in neurons (Fig. 3). ELISA assay was performed to evaluate the effect of ER stress induction on the secretion of endogenous Aβ1-40 and Aβ1-42 (Fig. 4). Golgi stress was induced by two different ionophores (i.e. monensin, nigericin), and immunocytochemical analysis showed a marked fragmentation of the Golgi apparatus and the localization of Syx5 was associated with the dispersed structure (Fig. 5). Finally, we provided the western blotting data that show the specific properties of Syx5 by comparing with the expression of other Syx family proteins in ER and Golgi stress induced neurons (Fig. 6).

### 1.1 Materials

Tunicamycin (Tm), Hoechst 33342, and brefeldin A (BFA) were purchased from Sigma-Aldrich Chemical Co. (St. Louis, MO); monensin and nigericin were obtained from Merck (Darmstadt, Germany). Monensin, nigericin, and BFA were dissolved in methanol. Staurosporine (STS) and thapsigargin (Tg) were also purchased from Merck and dissolved in dimethyl sulfoxide. A protease inhibitor cocktail was purchased from Wako Chemicals (Osaka, Japan). All other reagents were of the highest grade available, unless otherwise noted.

### 1.2 Antibodies

Mouse anti-Syx5 monoclonal antibody (clone 1C5) was prepared as described previously [4]. The preparation of other antibodies were described previously [3].

### 1.3 Hippocampal primary culture

Pregnant Wistar Kyoto rats were obtained from Japan SLC, Inc. (Hamamatsu, Shizuoka, Japan). All of the experimental procedures using animals were approved by the Experimental Animal Ethics Committee of the Kyorin University School of Medicine, and performed in accordance with the guidelines for handling laboratory animals. Primary rat hippocampal cultures were prepared as described previously [5]. In brief, hippocampi from postnatal day-0 pup brains were harvested in L15 medium (Sigma-Aldrich), dissociated in 2.5 mg/mL trypsin (DIFCO, Detroit, MI) and DNase (Sigma-Aldrich) for 15 min at 37 °C, and triturated with a siliconized pipette. Cells were plated at a density of 4–8×10^4^/cm^2^ in either a glass-bottomed 35-mm dish, in polyethyleneimine-coated 96-well plates (Greiner Bio-One, Frickenhausen, Germany), or in 12-well plates (Corning, NY) and cultured in DMEM (Sigma-Aldrich) containing 10% fetal bovine serum at 37 °C with 95% air, 5% CO_2_ in a humidified incubator. After 24 h of in vitro culture, the medium was replaced with serum-free, DMEM-containing GlutaMax and 2% B-27 Supplement (Life Technologies, Rockland, MD) either with or without 2 µM Ara-C (Sigma-Aldrich) and used after 13–16 days of in vitro culture.

### 1.4 Duplex siRNA

The sequences and preparation of Syx5 siRNA#1 (R424442) and control siRNA were described previously [3].

### 1.5 Extract preparation, SDS-PAGE, western blot analysis, and transfection

Biochemical analyses were carried out as described previously [3,6–8]. Transfection of annealed duplex siRNA (final concentration of 30 nM) was performed using Lipofectamine 2000 or annealed duplex siRNA (final concentration of 10 nM) was performed using Lipofectamine RNAiMax transfection reagents from Life Technologies.

### 1.6 Immunocytochemistry

Immunocytochemical analyses were carried out as described previously [3,8,9].

### 1.7 Ca^2+^ imaging

Hippocampal cells were treated with Fura2-AM (AM; acetoxy-methyl ester, Molecular Probes) for 25 min, washed, and allowed to stand for 20 min in normal medium. After changing the medium to HEPES containing Hank’s balanced salts solution, the intracellular Ca^2+^ concentration ([Ca^2+^]_i_) was measured. Fura2 ratio imaging analysis was performed using an ARGUS50 Ca^2+^ imaging system (Hamamatsu Photonics, Hamamatsu, Japan) and an inverted microscope (Daiphot300, Nikon, Japan) equipped with a CCD camera (C2400-80, Hamamatsu Photonics). Fluorescent images were taken at excitation wavelengths of 340 and 380 nm and were acquired every 10 s. Cells were treated with 1 μM Tg at the time shown by arrows. The ratio of Fura2 fluorescence (F340 nm/F380 nm) was calculated and plotted against time, and the values from single cells were averaged at each time point and expressed as the mean±SD (*n*=10). SERCA inhibitor, Tg induced a transient increase in [Ca^2+^]_i_.

### 1.8 Cell viability and cytotoxicity assays

Cell viability and cytotoxicity of cells were measured with a CellTiter-Glo Luminescent Cell Viability Assay and CytoTox-Glo Cytotoxicity Assay systems (Promega) according to the manufacturer’s instructions. Luminescence was measured using a luminometer (GloMax; Promega).

### 1.9 Quantification of rat Aβ peptides by sandwich enzyme-linked immunosorbent assay

Hippocampal neurons were cultured in 12-well plates (in 2 mL of medium) for 13 days before treatment. After the medium was replaced with fresh medium, the cells were treated with monensin or nigericin for 16 h. The culture medium was collected, and cell debris was removed by centrifugation at 15,000*g* for 10 min; the resultant supernatant fraction was used as the enzyme-linked immunosorbent assay (ELISA) sample. Aβ peptides secreted into the culture medium (100 μL) were quantified using a sandwich ELISA specific for either rat Aβ1-40 or Aβ1-42 according to the manufacturer’s instructions (Takara Bio).

### 1.10 Data analysis and statistics

The enhanced chemiluminescence signals from western blot analyses and the amount of each PCR product were analyzed using MultiGauge software (FujiFilm, Tokyo, Japan). Statistical analyses were performed using GraphPad Prism software (GraphPad Software, La Jolla, CA). Data are presented as mean±SEM unless noted. The number of samples examined is indicated in an inset within each figure. A *t*-test or ANOVA was used to determine the statistical significance of differences between values.

Data for Fig. 1. Hippocampal neurons plated in polyethyleneimine-coated 96 well plates were treated with vehicle or indicated concentration of STS (A), Tm (B), and Tg (C) for 16 h. Cell viability assay was performed as described in Materials and Methods. Each value is represented as a mean±S.D. (*n*=11-12). Data sets were subjected to one-way ANOVA and with Dunnet’s post-hoc tests, ***P<0.0001.

Data for Fig. 2. (A) Hippocampal neurons were subjected to Ca^2+^ imaging as described in Materials and Methods. Cells were treated with 1 μM Tg at the time shown by an arrow. Ratio of Fura2 fluorescence (F340 nm/F380 nm) were calculated and were plotted against time and the values from single cell were averaged at each time point and expressed as mean±SEM (*n*=10). SERCA inhibitor Tg induced transient increase in [Ca^2+^]_i_ but returned to the basal level of [Ca^2+^]_i_. (B) Hippocampal neurons were treated with siRNA for Syx5 (R424442) and cell extracts were subjected to SDS-PAGE and western blotting with anti-Syx5 (1C5). Representative images are shown. (C) Densitometric analysis of western blotting in (B). The amount of each Syx5 isoforms was quantified, and the values are expressed as the mean percentage of the control siRNA-treated cells (*n*=28). * *P*<0.05 and *** *P*<0.0001 *vs.* control, as determined by a *t*-test.

Data for Fig. 3. (A) Hippocampal neuron cultures were treated with 2 µg/mL brefeldin A (BFA), another ER stress inducer, for 16 h, and the cell extracts from 14DIV neurons were subjected to SDS-PAGE and western blot analysis. Western blots showing amounts of Syx5 isoforms were analyzed densitometrically. A significant increase in Syx5 expression was observed in BFA-treated cells. (B) RT-PCR analysis of neurons treated with 2 µg/mL of BFA. Transcripts were quantified, and the values are expressed as the mean ratio of measurements of the experimental cells to those of the vehicle-treated control cells. * *P*<0.05 and ** *P*<0.001 *vs.* vehicle control, as determined by a *t*-test. BFA treatment also induced *de novo* syntheses of ER–Golgi SNARE Syx5. (C) Effect of ER stress inducers on the intracellular localization of Syx5 isoforms and on the morphology of the Golgi apparatus. 16DIV hippocampal neurons were fixed and stained for Syx5 or GM130 after exposure to 1 µM Tg or 2 µg/mL BFA for 16 h. Representative images are shown. Tg and BFA treatments induced fragmentation of the Golgi as assessed by the localization of Golgi matrix protein GM130 (green). Dispersion of the Golgi apparatus observed in BFA-treated cells shows that BFA had a stronger effect than Tg on Golgi structure. Blue, nuclei stained with Hoechst 33342; red, Syx5 stained with monoclonal antibody 1C5 with a Cy3-conjugated secondary antibody; green, Golgi apparatus stained with monoclonal antibody against Golgi marker GM130 with an Alexa Fluor 488-conjugated secondary antibody.

Data for Fig. 4. Effect of ER stress inducers on Aβ peptide secretion was examined. (A). Hippocampal neurons were treated with 1 µM Tg or 2 µg/mL BFA for 16 h. Cell extracts were prepared 16 hours after the treatment and subjected to SDS-PAGE and western blotting with anti-βAPP (APPC; 1:2,000) antibodies. A representative image from 9 independent samples is shown. (B) The amount of intracellular βAPP holoprotein was quantified densitometrically (*n*=9). Significant accumulation of endogenous βAPP holoproteins was observed in BFA-treated, but not in Tg-treated cells as in NG108-15 cells [3]. (C) Endogenous rat Aβ1-40 and Aβ1-42 peptides secreted from the cells during 16 h of treatment with ER stressors was quantified by selective sandwich ELISA, as described in Section 1. The amount of each peptides is expressed as the mean±SEM percent relative to that of the vehicle (*n*=12). ***P<0.001 *vs.* vehicle, as determined by a *t*-test. Secretion of both Aβ peptides was markedly suppressed by induction of ER stress.

Data for Fig. 5. Nigericin and monensin treatment induced fragmentation of the Golgi. 14DIV neurons cultured in poly-L lysine-coated glass-bottomed 35 mm dish were treated with 0.1 µM nigericin and 1 µM monensin for 16 h. Then cells were fixed and stained for GM130 or Syx5. Representative images are shown. Blue; nuclei stained with Hoechst 33342. Red; Golgi apparatus stained with monoclonal antibody to Golgi matrix protein GM130 or Syx5 stained with monoclonal antibody 1C5 with Alexa Fluor 555-conjugated secondary antibody. Nigericin and monensin induced marked Golgi fragmentation and distribution of Syx5 was dispersed in hippocampal neurons.

Data for Fig. 6. (A) Hippocampal neuron cultures were treated with 1 µM Tg (*n*=26) or 2 µg/mL BFA (*n*=9) for 16 h, and the cell extracts were subjected to SDS-PAGE and western blot analysis. Western blots showing amounts of indicated Syxs were analyzed densitometrically (B). (C) Culture neurons were treated with 0.1 µM nigericin or 1 µM monensin for 16 h, and the cell extracts were subjected to SDS-PAGE and western blot analysis. Expressed amounts of Syx6 and Syx1A were analyzed densitometrically (D). *P<0.05 *vs.* vehicle control, as determined by a *t*-test (*n*=10–14). Among Syxs examined, both ER and Golgi stressors did not induce significant increase in the expression of Syx6 nor Syx1A.
